# Pharmacological inhibition of mTOR attenuates replicative cell senescence and improves cellular function via regulating the STAT3-PIM1 axis in human cardiac progenitor cells

**DOI:** 10.1038/s12276-020-0374-4

**Published:** 2020-04-09

**Authors:** Ji Hye Park, Na Kyoung Lee, Hye Ji Lim, Seung taek Ji, Yeon-Ju Kim, Woong Bi Jang, Da Yeon Kim, Songhwa Kang, Jisoo Yun, Jong seong Ha, Hyungtae Kim, Dongjun Lee, Sang Hong Baek, Sang-Mo Kwon

**Affiliations:** 1 Laboratory of Regenerative Medicine and Stem Cell Biology, Department of Physiology, Medical Research Institute, School of Medicine, Pusan National University, Yangsan, 50612 Republic of Korea; 2 Research Institute of Convergence Biomedical Science and Technology, Pusan National University School of Medicine, Yangsan, 50612 Republic of Korea; 3 R&D Center for Advanced Pharmaceuticals & Evaluation, Korea Institute of Toxicology, Korea Research Institute of Chemical Technology, 141 Gajeong-ro, Yuseong-gu, Daejeon, 34114 South Korea; 4 Department of Thoracic and Cardiovascular Surgery, Pusan National University Yangsan Hospital, School of Medicine, Pusan National University, Yangsan, 50612 Republic of Korea; 5 Department of Convergence Medical Science, Pusan National University School of Medicine, Yangsan, 50612 Republic of Korea; 6 Laboratory of Cardiovascular Disease, Division of Cardiology, School of Medicine, The Catholic University of Korea, Seoul, 137-040 Republic of Korea

**Keywords:** Adult stem cells, Heart stem cells

## Abstract

The mammalian target of rapamycin (mTOR) signaling pathway efficiently regulates the energy state of cells and maintains tissue homeostasis. Dysregulation of the mTOR pathway has been implicated in several human diseases. Rapamycin is a specific inhibitor of mTOR and pharmacological inhibition of mTOR with rapamycin promote cardiac cell generation from the differentiation of mouse and human embryonic stem cells. These studies strongly implicate a role of sustained mTOR activity in the differentiating functions of embryonic stem cells; however, they do not directly address the required effect for sustained mTOR activity in human cardiac progenitor cells. In the present study, we evaluated the effect of mTOR inhibition by rapamycin on the cellular function of human cardiac progenitor cells and discovered that treatment with rapamycin markedly attenuated replicative cell senescence in human cardiac progenitor cells (hCPCs) and promoted their cellular functions. Furthermore, rapamycin not only inhibited mTOR signaling but also influenced signaling pathways, including STAT3 and PIM1, in hCPCs. Therefore, these data reveal a crucial function for rapamycin in senescent hCPCs and provide clinical strategies based on chronic mTOR activity.

## Cardiac regeneration: preventing premature ageing

Disrupting the mTOR signaling pathway, a major regulator of cell growth, protects human cardiac progenitor cells (hCPCs) from irreversible cell cycle arrest, also known as replicative cell senescence. Accumulation of senescent (aging) hCPCs leads to premature cardiac ageing and heart failure by hindering tissue repair in response to damage. Previous studies have shown that the mTOR inhibitor rapamycin promotes the differentiation of embryonic stem cells into hCPCs, but little is known about its effects on senescent hCPCs. Ji Hye Park at Pusan National University in South Korea and colleagues found that rapamycin reduced the expression of senescence-related markers in hCPCs and improved the ability of hCPCs to proliferate, migrate, and differentiate into cardiac muscle cells. These findings suggest that rapamycin could be used to aid cardiac regeneration following heart muscle damage caused by a heart attack.

## Introduction

Aging is associated with an organism progressively losing the ability to maintain homeostasis and repair tissue damage^1^. The predominant aging mechanism occurs because of the accumulation of senescent cells in various tissues and organs^2^. These senescent cells have undergone an irreversible cell cycle arrest^3^ and are characterized by the expression of *p16*^*Ink4a*^^4^. The prevalence of cardiovascular diseases increases with aging. Investigators have demonstrated that the number of the left ventricle cardiomyocytes progressively declines with aging^5^. Several studies have reported that undifferentiated primitive cells reside in mammalian hearts and protect them against heart failure^1,6,7^. Furthermore, senescent and dysfunctional resident human cardiac progenitor cells (hCPCs) accumulate as a consequence of cardiac pathology^8–12^ and lead to premature cardiac aging and heart failure^13^. These hCPCs act as key regulators of cardiomyocyte homeostasis in the heart^14,15^ and contribute to the repair of damaged heart tissue^16^.

The mammalian target of rapamycin (mTOR) is a serine–threonine protein kinase that regulates vital processes, including protein synthesis, autophagy, cell growth, metabolism, and survival^17–20^. mTOR exists in two independent multiprotein-containing complexes (mTORC1 and mTORC2), which have distinct functions in the adult tissue^21^. mTOR plays an indispensable role in embryonic stem cell maintenance, embryonic heart development, and adult heart homeostasis^22–24^ during cardiogenesis. In addition, *mtor* knockout mice were shown to exhibit heart failure and dilated cardiomyopathy^25,26^. Rapamycin is a specific inhibitor of mTOR and is known to be useful in treating diseases such as cancer, diabetes, obesity, neurological diseases, and genetic disorders^27^. Recent studies demonstrated that rapamycin is an mTORC1 antagonist^28–30^ that can also inhibit mTORC2 activity in some cell types^31^. The other ATP-competitive inhibitors of mTOR, namely, PP242, have recently been demonstrated to have more potent antileukemic activity than rapamycin^32^. In addition, rapamycin can efficiently promote cardiac cell generation from the differentiation of mouse embryonic stem cells^33,34^. These observations indicate that chronic mTOR activity is important for the differentiation of embryonic stem cells into cardiac cells; however, the role of chronic mTOR activity in hCPC regulation remains unclear.

In this study, we demonstrated that mTOR inhibition by rapamycin markedly attenuated replicative cell senescence in hCPCs and promoted cellular functions such as proliferation, migration, clonogenicity, and differentiation. Moreover, rapamycin not only inhibited mTOR signaling but also influenced the STAT3-PIM1 signaling pathway in hCPCs. Collectively, our data reveal the crucial function of rapamycin in senescent hCPCs, which could be important for developing novel therapeutic interventions.

## Materials and methods

### Human cardiac progenitor cell isolation and culture

c-Kit^+^ hCPCs were isolated from infant heart tissue, as previously described^16^. The study was approved by the Ethics Review Board of Pusan National University Yangsan Hospital, Gyeongsangnam-do, Republic of Korea (IRB 05-2015-133). Human cardiac tissues were first mechanically disaggregated with 0.2% collagenase type II (Warthington Biochemical, Corp., Lakewood, NJ, USA). Single cardiac cells were incubated and expanded in cardiac expansion media. When the cells reached 70–80% confluence, the cells were incubated with a c-Kit primary antibody (Santa Cruz Biotechnology, Santa Cruz, CA, USA) and a secondary rabbit-IgG bead. Furthermore, the c-Kit^+^ cells were sorted via magnetically activated cell sorting. In this study, young hCPCs (passage numbers < 8) were used as control cells and senescent hCPCs (passage numbers > 16) were used as senescent hCPCs.

### Rapamycin treatment

hCPCs were cultured in Ham’s F12 medium (Hyclone, GE Healthcare, Chicago, IL, USA) comprising 10% fetal bovine serum (FBS; Gibco, Thermo Fisher Scientific, Carlsbad, CA, USA), 1% penicillin–streptomycin (Welgene, Daegu, Republic of Korea), 5 µg of recombinant human basic fibroblast growth factor (Peprotech, Rocky Hill, NJ, USA), 2.5 U of human erythropoietin (R&D Systems, Minneapolis, MN, USA), and 2 mM glutathione (Sigma-Aldrich). Rapamycin (Sigma-Aldrich, St. Louis, MO, USA) treatment typically started at passage 7 for the experiments. Various concentrations (1 nM, 10 nM, and 100 nM) of rapamycin were added to the hCPC medium and the medium was replaced every 2 days. A similar amount of dimethyl sulfoxide (DMSO) that was utilized to treat hCPCs was used as a control.

### Cell proliferation assay

The cell proliferation assay was performed using an MTS kit (EzCytox, Dail Tech Seoul, Korea) according to the manufacturer’s instructions. Cell proliferation of hCPCs following treatment with rapamycin (0, 1, 10, and 100 nM) was tested via a Bromodeoxyuridine (BrdU) cell proliferation assay kit (Cell Signaling Technology). Each experiment was repeated three times.

### Immunoblotting analysis

Total lysates from human hCPCs were prepared using radioimmunoprecipitation assay buffer (Thermo Scientific, Rockford, IL, USA) and were then used for western blotting. Proteins were separated via SDS-polyacrylamide gel electrophoresis and were then electrotransferred onto polyvinylidene difluoride membranes (Millipore). The membranes then were blocked with 5% skim milk in Tris-buffered saline with 0.1% Tween 20 (TBS-T) for 1 h at room temperature. Thereafter, the membranes were incubated overnight with primary antibodies at 4 °C. Antibodies were used against p16 (1:1000, Abcam), p21 (1:1000, Santa Cruz), p53 (1:1000, Abcam), STAT3 (1:1000, Cell Signaling Technology), p-STAT3 (1:500, Cell Signaling Technology), Pim1 (1:1000, Abcam), and GAPDH (1:2000, Santa Cruz). Membranes were washed with TBS-T and were incubated with a peroxidase-conjugated secondary antibody. The bands were visualized via LAS 3000 (Fujifilm).

### Senescence-associated β-gal (SA β-gal) assay

To compare the senescence-associated β-gal (SA-β-gal) activity between the control and senescent cells, and to examine whether rapamycin promotes SA-β-gal activity long term in senescence, hCPCs were treated with rapamycin (0, 1, 10, and 100 nM). Moreover, SA-β-gal activity was measured with a SA-β-gal kit (Cell Signaling Technology) according to the manufacturer’s instructions. SA-β-gal-positive cells were quantified by counting the number of cells in ten random microscopic fields per filter (×200 magnification).

### Migration assay

To compare the migration ability of cells, hCPCs were seeded at a density of 2 × 10^5^ cells on a 12-well plate (Thermo Scientific) and they were grown until they attained 100% confluence. Using a sterile yellow pipette tip, a scratch was created in each well. After 8 h of incubation, the wound distance of the cells was visualized at ×40 magnification using a light microscope (Olympus). For transwell migration assays, DMSO (young: using less than passage 8, senescent: using greater than passage 17) and long-lasting treated hCPCs (passage 17) were suspended in serum-free F12 Ham’s medium and placed in 8 μm pore inserts (Corning) to migrate toward complete hCPC culture medium. After 24 h of incubation, the cells were removed from the medium and fixed at room temperature for 10 min with 4% paraformaldehyde. The membrane was stained at room temperature for 30 min with 0.005% crystal violet in 20% MeOH solution. Upper surface unmigrated cells were removed by a cotton swab. The cell migration ability was quantified by enumerating the cell count in 10 random 200 × microscopic fields.

### Tube formation assay

The capability of hCPCs to form a capillary-like structure was assessed via Matrigel assays. hCPCs (8 × 10^3^ cells) were seeded onto a 96-well plate (Thermo Scientific) coated with growth factor-reduced Matrigel (55 µl/well, BD Biosciences). Cells were incubated for longer than 6 h at 37 °C in 5% CO_2_. After incubation, the total number of tubes was determined using ImageJ software.

### Clonogenic assay

To test clonogenicity, single hCPCs were seeded into 96-well gelatin-coated plates by serial dilution and the medium was changed every 3 days. After 7 days, the clones were identified and expanded. Cells were grown for 2 weeks. The clonogenicity of hCPCs was calculated by counting the number of clones per well and then that number was divided by the total number of wells.

### Quantitative real-time PCR

Complete RNA was extracted using TRIzol reagent (Invitrogen) according to the manufacturer’s instructions. cDNA was synthesized using the PrimeScript^TM^ 1st strand cDNA synthesis kit (Takara Biotechnology). Quantitative PCR was performed using a SYBR green PCR master mix on the Light Cycler 96 instrument (Roche). The sequence of the primers used in this study is listed in the Supplementary Material.

### RNA-sequencing

Approximately 2 µg of total RNA was isolated, purified, and fragmented as mRNA. RNA-sequencing (RNA-seq) libraries were constructed by Teragen Bio Institute according to the manufacturer’s specifications. Differentially expressed gene (DEG) analysis was performed based on the Cuffdiff method^35^. DEG analysis was based on the *q*-value threshold of <0.05 for rectifying the errors caused by multiple testing. A Gene Ontology (GO) database was created based on the biological process (BP), cellular component (CC), and molecular function (MF).

### Differentiation assay

To evaluate the smooth muscle cell (SMC) differentiation ability, hCPCs were treated with Medium 231 (Gibco) supplemented with 5% FBS and smooth muscle differentiation supplement (Gibco) for 7 days. The medium was changed every 2 days. To investigate the cardiomyocyte differentiation ability, hCPCs were treated with MEM/EBSS medium (HyClone) supplemented with 2% FBS and 10 nM dexamethasone for 28 days. The medium was changed every day.

### Statistical analysis

The sample sizes required for the experiments were estimated based on the preliminary results. No blinding or randomization was performed for any of the experiments. All data are presented as the mean ± SEM. The experimental control and treated groups were analyzed using a two-tailed unpaired Student’s *t*-test, unless otherwise indicated. Statistically significant differences (*p ≤ 0.05, **p ≤ 0.01, ***p ≤ 0.001) for pairwise comparisons between the experimental control and treated groups are presented in this study.

## Results

### Characterization of replicative senescent hCPCs

To characterize the phenotype of replicative senescence in hCPCs, we maintained the cells over 16 passages to generate spontaneous replicative senescence (Supplementary Fig. S1). In accordance with previous studies on cellular replicative senescences^2–4^, the length and width of the cells and the SA-β-gal^+^ activity were increased in senescent hCPCs (Supplementary Fig. S1A, B) compared with that of young controls (less than eight passages). In addition, the expression of both cyclin-dependent kinase inhibitors and senescence-associated markers, such as p21 and p16, was increased in the senescent hCPCs (Supplementary Fig. S1C, D). Furthermore, to address the function of replicative senescence in hCPCs, we evaluated the proliferation, migration, and tube formation abilities of senescent hCPCs (Supplementary Fig. S1E–G). These abilities of the capillary network were significantly reduced in the senescent hCPCs compared with the control of young cells (Supplementary Fig. S1E–G). Furthermore, to determine whether the transcriptome program was altered in the senescent hCPCs, we analyzed their transcripts via RNA-seq (Supplementary Fig. S1H). We found that 2249 and 2420 transcripts were upregulated and downregulated in senescent hCPCs, respectively, including genes that regulate the cell cycle and DNA replication. These data suggest that we initially generated cellular replicative senescence in the hCPCs and characterized the molecular and physiological phenotype of replicative senescence in hCPCs.

### Effects of prolonged rapamycin treatment in senescent hCPCs

Several studies have reported that rapamycin efficiently promotes cardiac cell generation from the differentiation of mouse embryonic stem cells^33,34^. These observations indicate that chronic mTOR activity is important for the differentiation function of embryonic stem cells; however, the role of chronic mTOR activity in senescent hCPCs remains unclear.

**Fig. 1:**
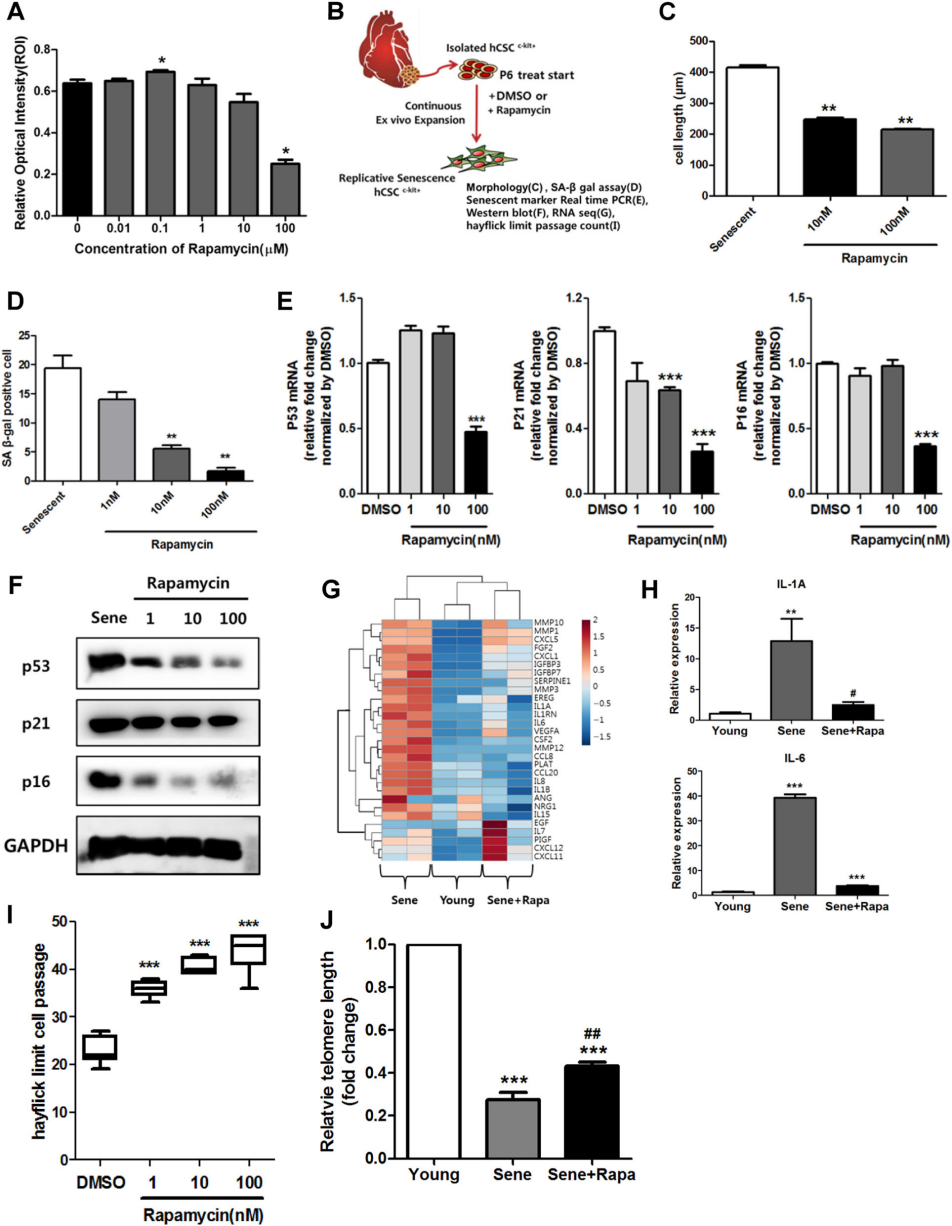
Rapamycin attenuated hCPC senescence. **a** A cytotoxicity assay was used to assess senescent hCPCs after prolonged rapamycin treatment using an MTS kit (*p ≤ 0.05, *n* = 8). **b** Scheme of experiments. **c** Quantification of the length of senescent hCPCs after prolonged rapamycin treatment (**p ≤ 0.01, *n* = 10). **d** Quantification of SA-β-gal staining after prolonged treatment with different concentrations of rapamycin. **e**, **f** Measurement of the expression of the following senescent markers: p53, p21, and p16 (***p ≤ 0.001, *n* = 3). **g** Heat map of SASP-related genes. **h** Expression levels of IL1A and IL6 were analyzed in senescent hCPCs after prolonged rapamycin treatment using qRT-PCR (**p ≤ 0.01, *n* = 3). **i** A count of the Hayflick passage numbers in the senescent hCPCs after prolonged rapamycin treatment (***p ≤ 0.001, *n* = 6). **j** Measurement of the relative telomere length in the senescent hCPCs after prolonged rapamycin treatment (***p ≤ 0.001, vs. young, ^##^ ***p ≤ 0.01, vs. senescent, *n* = 3). Error bars indicate SEM.

**Fig. 2:**
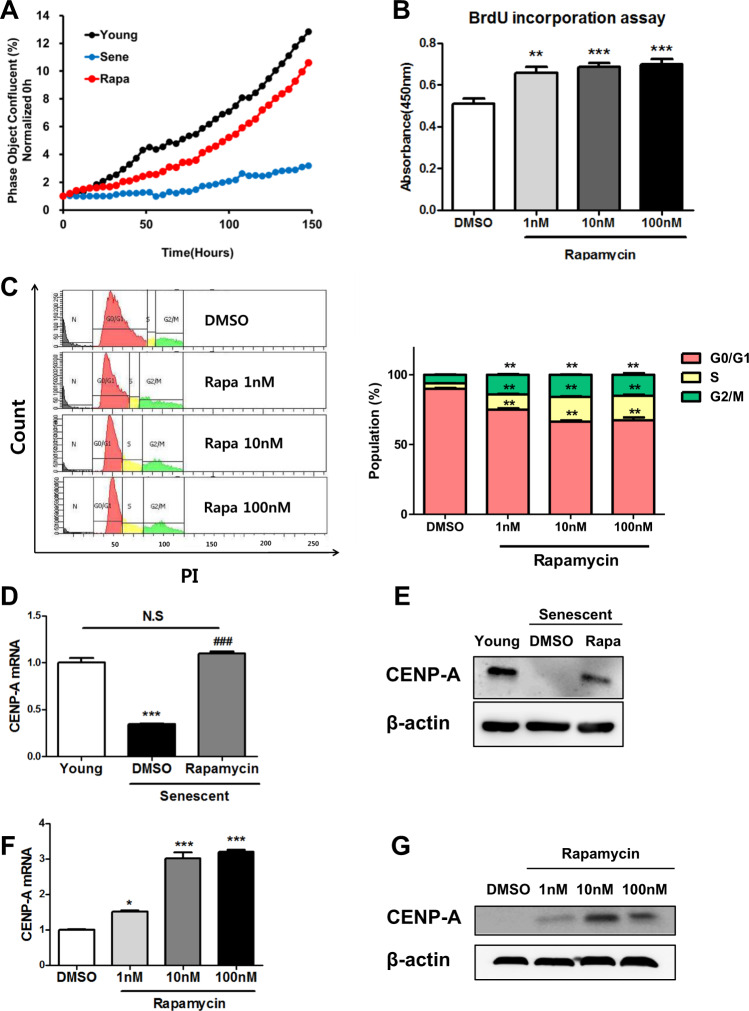
Rapamycin improved senescent hCPC proliferation. **a** Real-time cell proliferation analysis (young passage number 8; senescent passage number 18; rapamycin passage number 18). **b** Cell proliferation assay was detected by BrdU labeling after prolonged treatment with different concentrations of rapamycin (**p ≤ 0.01, ***p ≤ 0.001, *n* = 6). **c** Cell cycle analysis (**p ≤ 0.01, *n* = 0.03). **d**, **g** The expression of CENP-A from senescent hCPCs after prolonged rapamycin treatment (*p ≤ 0.05, ***p ≤ 0.001, *n* = 3). Error bars indicate SEM.

**Fig. 3:**
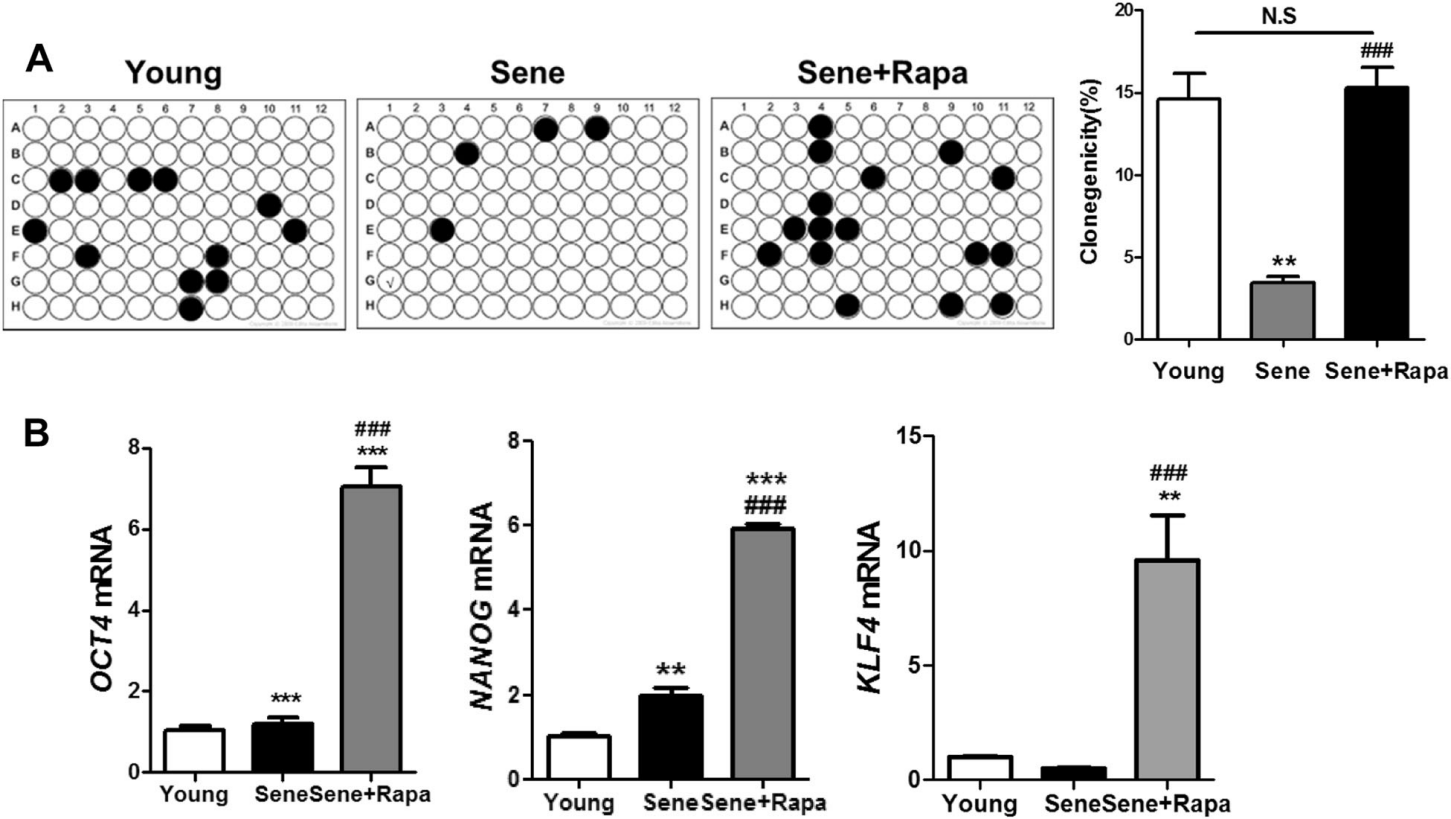
The effect of rapamycin on the clonogenic potential of senescent CPCs. **a** Clonogenic ability in senescent hCPCs after prolonged rapamycin treatment. The graph represents the percentage of wells that maintained clones for 14 days (**p ≤ 0.01, vs. young, ###*p* ≤ 0.001, vs. senescent). **b** The expression of the clonogenicity markers (OCT4, KLF4, and NANOG) was examined in senescent hCPCs after prolonged rapamycin treatment via qRT-PCR (*p ≤ 0.05). Error bars indicate SEM.

**Fig. 4:**
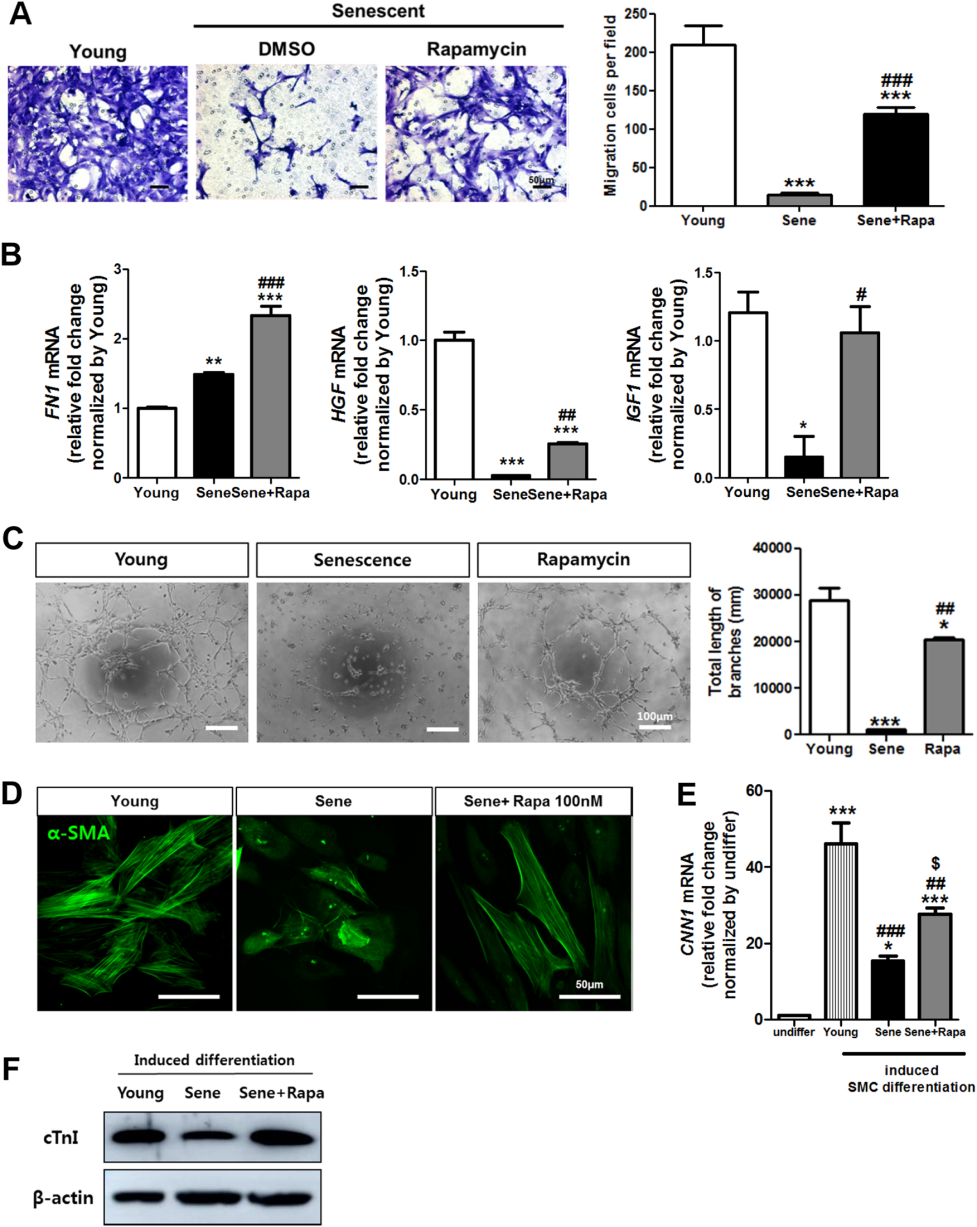
The effect of rapamycin on the migration and tube formation ability of senescent CPCs. **a** The migration ability of the senescent hCPCs was evaluated after prolonged rapamycin treatment via transwell migration assays. **b** The expression of cell migration-related markers such as FN1, HGF, and IGF1 was assessed in senescent hCPCs after prolonged rapamycin treatment by qRT-PCR (**p ≤ 0.01, vs. young, ##*p* ≤ 0.01, vs. senescent, *n* = 3). **c** Tube formation ability was assessed in the young and senescent hCPCs after prolonged rapamycin treatment by Matrigel tube formation assay (***p ≤ 0.001 vs. young, #*p*, vs. senescent, *n* = 3). **d** Immunofluorescence was performed with antibodies against smooth muscle α-actin (α-SMA). **e** Analysis of smooth muscle cell marker *CNN1* in young and senescent hCPCs after prolonged rapamycin treatment by qRT-PCR (**p ≤ 0.01, vs. undifferentiated, ***p ≤ 0.001, vs. undifferentiated, #*p*, vs. young, *$p*, vs. senescent, *n* = 3). **f** Expression of cTnI after induced myogenic differentiation was investigated by western blotting. Error bars indicate SEM.

**Fig. 5:**
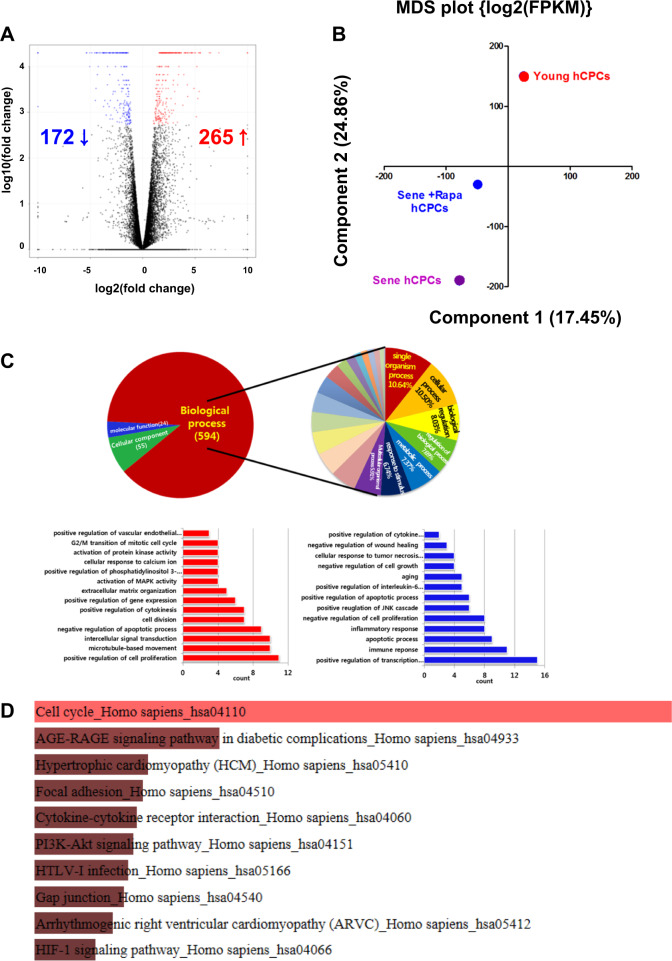
Gene Ontology (GO) term enrichment analysis of transcripts regulated by prolonged treatment with rapamycin. **a** A volcano plot was presented between the senescent hCPCs before and after prolonged rapamycin treatment. **b** Principal component analysis (PCA) of 18,001 genes with FPKM > 0 in senescent hCPCs after prolonged rapamycin treatment. **c** The pie chart presents the senescent hCPCs after prolonged rapamycin treatment. Left lower panel: Upregulated GO biological process is indicated in the senescent hCPCs after prolonged rapamycin treatment. Right lower panel: Downregulated GO biological processes were revealed in senescent hCPCs after prolonged rapamycin treatment. **d** KEGG pathway enrichment analysis. Top ten enriched pathways in the KEGG database are presented in the bar graph.

**Fig. 6:**
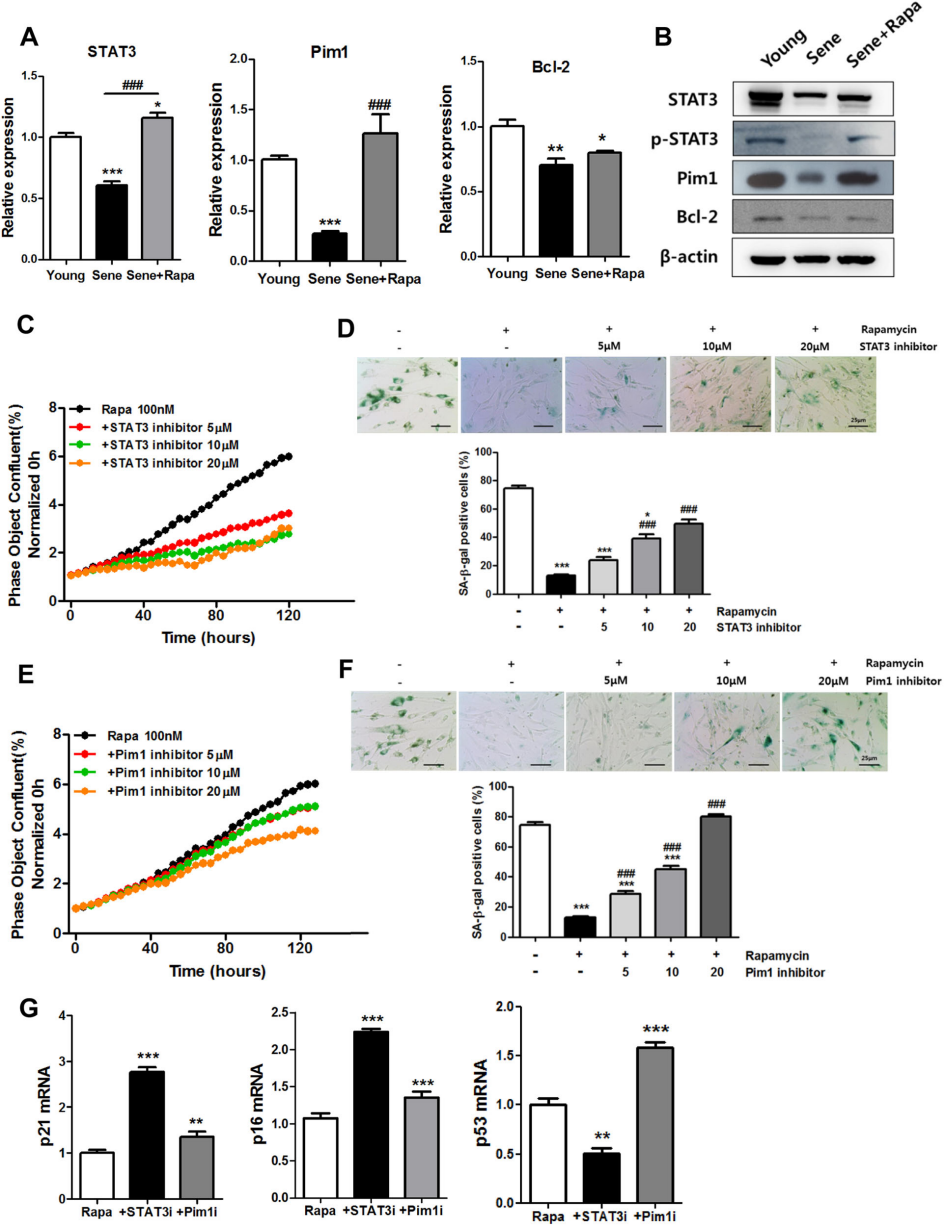
The roles of STAT3-PIM1 in the rapamycin-induced rescue of CPC senescence. **a**, **b** Expression of *STAT3*, *Pim1*, and *Bcl-2* was examined in senescent hCPCs after prolonged rapamycin treatment (*p ≤ 0.05, **p ≤ 0.01, vs. young, ###*p* ≤ 0.001, vs. senescent, *n* = 3). **c** Proliferation assay of cells treated with the STAT3 inhibitor S31–201 in senescent hCPCs after prolonged rapamycin treatment. **d** Senescence β-gal assay on treatment with STAT3 inhibitor S31–201 in senescent hCPCs after prolonged rapamycin treatment (*p ≤ 0.05, ***p ≤ 0.001, vs. senescence hCPCs, ###, ***p ≤ 0.001 *n* = 3). **e** Proliferation assay following treatment of senescent hCPCs with the PIM1 inhibitor after prolonged rapamycin treatment. **f** Senescence β-gal assay on following treatment with the PIM1 inhibitor S31–201 in senescent hCPCs after prolonged rapamycin treatment. **g** The expression of senescence markers p21, p16, and p53 was examined by qRT-PCR after treatment with STAT3 and Pim1 inhibitors (**p ≤ 0.05, ***p ≤ 0.001, vs. prolonged rapamycin-treated hCPCs). Abbreviations: STAT3i STAT3 inhibitor, Pim1i Pim1 inhibitor. Error bars indicate SEM (*p ≤ 0.05, **p ≤ 0.01, *n* = 3).

Initially, to investigate rapamycin cytotoxicity in senescent hCPCs, we performed MTS (3-(4,5-dimethylthiazol-2-yl)-5-(3-carboxymethoxyphenyl)-2-(4-sulfophenyl)-2H-tetrazolium) assays in senescent hCPCs after treating them with different concentrations of rapamycin (Fig. 1a); with 1 µM rapamycin, no significant difference was observed in cell viability in senescent hCPCs. Thus, we selected 100 nM as the concentration of rapamycin for the following experiments (Fig. 1–6). Furthermore, to address the effects of prolonged rapamycin treatment on senescent hCPCs, we continually treated cells with rapamycin during passaging (Fig. 1b). The cell length, SA-β-gal^+^ activity, and the expression of senescence-associated markers (p53, p21, and p16) were significantly decreased in the senescent hCPCs after prolonged rapamycin treatment (Fig. 1c–f). The major feature of senescent cells is the secretion of cytokines and growth factors, which is termed the senescence-associated secretory phenotype (SASP)^36^. The expression of genes related to SASP were increased in the senescent hCPCs as shown by column clustered heat maps (Fig. 1g). In these hCPCs, after prolonged rapamycin treatment, the expression of genes related to SASP was decreased to levels similar to those in the young control hCPCs. The expression of SASP components, such as IL1A and IL6, in the senescent hCPCs after prolonged rapamycin treatment was significantly decreased compared with that of the senescent hCPCs (Fig. 1h).

The Hayflick limit is defined as the number of times a cell population will divide before cell division stops^37^. The Hayflick limit passage was significantly increased in the hCPCs after prolonged rapamycin treatment (Fig. 1i). Moreover, the telomeres are shortened or eliminated during replicative cellular senescence and cell cycle arrest^38^. Prolonged rapamycin treatment contributed to increased telomere length (Fig. 1j). Collectively, these data suggest that rapamycin is a potential attenuating agent for replicative senescent hCPCs.

### Effects of prolonged rapamycin treatment on the proliferation of senescent hCPCs

To evaluate the proliferative effect of prolonged rapamycin treatment on hCPCs, we performed real-time cell proliferation (Fig. 2a) and BrdU (Fig. 2b) assays. The proliferation rate significantly increased in the senescent hCPCs after prolonged rapamycin treatment compared with the levels observed before the treatment (Fig. 2a, b). Thereafter, we tested whether prolonged rapamycin treatment contributed to cell cycle progression by flow cytometric analysis (Fig. 2c) and found that it caused the accumulation of the S/G2/M phase in senescent hCPCs.

Centromere protein A (CENP-A) is one of the important components in the cell cycle and it functions in mitotic spindle assembly^39^. Several studies demonstrated that CENP-A is crucial for CPC proliferation, and that it inhibits senescence and promotes survival following differentiation^40^. In addition, the expression of CENP-A is decreased in the CPCs from aged mice^40^. In accordance with a previous study on CENP-A expression^40^, the expression of CENP-A was decreased in replicative senescent hCPCs (Fig. 2d, e) in this study. Interestingly, the expression of CENP-A in senescent hCPCs after prolonged rapamycin treatment was increased to levels that were similar to those of the young hCPC controls (Fig. 2d, e). Furthermore, the expression of CENP-A in the senescent hCPCs after prolonged rapamycin treatment increased in a rapamycin dose-dependent manner (Fig. 2f, g). Collectively, these data indicate that rapamycin promotes the proliferation of senescent hCPCs.

### Effects of prolonged rapamycin treatment on the clonogenic potential and migration/differentiation of senescent hCPCs

To determine whether prolonged rapamycin treatment affected stem cell properties in senescent hCPCs, we generated single-cell-derived clones (Fig. 3a). Clonogenic potential was decreased in the senescent hCPCs and it was increased in the senescent hCPCs after prolonged rapamycin treatment. Furthermore, we evaluated the expression of cardiac progenitor cell surface marker proteins from senescent hCPCs after prolonged rapamycin treatment (Supplementary Fig. S2). No differences were observed in the expression of CD44, CD105, CD166, and CD29 in the senescent hCPCs before and after prolonged rapamycin treatment (Supplementary Fig. S2). These data suggest that prolonged rapamycin treatment does not affect the expression of surface markers in hCPCs. To address the question of whether rapamycin affects gene expression to regulate clonogenic potential, we assessed the expression of *OCT4*, *NANOG*, and *KLF4* in senescent hCPCs after prolonged rapamycin treatment. Interestingly, the expression of these genes was significantly increased in senescent hCPCs after prolonged rapamycin treatment (Fig. 3b), which indicated that this treatment improved the senescence clonogenic ability.

The migration ability of hCPCs is essential for homing and amelioration of myocardial injury. Recent studies have reported that fibronectin induces the migration/differentiation of hCPCs^41^, and HGF and IGF1 are crucial for the regeneration of CPC-mediated cardiac injury^42,43^. The migration activity and expression of FN1, HGF, and IGF1 were increased in the senescent hCPCs after prolonged rapamycin treatment (Fig. 4a, b).

The hCPCs can differentiate into endothelial cells and SMCs^44^. First, to assess whether prolonged rapamycin treatment affects the differentiation activity in endothelial cells, we performed a tube formation assay using senescent hCPCs. The total tube length and number of branches were significantly increased in the senescent hCPCs after prolonged rapamycin treatment (Fig. 4c). Second, to test whether prolonged rapamycin treatment affects differentiation activity in SMCs, we induced SMC differentiation. Immunofluorescence analysis revealed that the expression of α-smooth muscle actin (α-SMA) is increased in senescent hCPCs after prolonged rapamycin treatment (Fig. 4d). In addition, the expression of SMC markers, such as *CNN1*, was significantly increased in rapamycin-treated hCPCs compared with senescent hCPCs (Fig. 4e). Next, we assessed hCPC myogenic differentiation ability. The differentiation into myocardial cells was verified by the expression of the cardiomyocyte filament proteins cardiac troponin (cTnI). As shown in Fig. 4f, cTnI expression was decreased in senescent hCPCs. In contrast, the expression of cTnI increased in rapamycin-treated hCPCs. Collectively, these data suggest that prolonged rapamycin treatment enhances the migration ability and endothelial/SMC/cardiomyogenic differentiation ability of senescence hCPCs.

### Molecular mechanism of prolonged rapamycin treatment in senescent hCPCs

To further understand the impact of prolonged rapamycin treatment on hCPC senescence, we performed RNA-seq analysis. A volcano plot of gene expression analysis demonstrated that 265 genes were upregulated and 172 genes were downregulated in senescent hCPCs after prolonged rapamycin treatment when compared with cells before treatment (Fig. 5a). Principal component analysis revealed that rapamycin-treated hCPCs were more closely positioned to young hCPCs. Furthermore, we performed GO enrichment analyses (cutoff, *p* < 0.001) to identify the rapamycin-dependent genes and pathways involved in the senescent hCPCs (Fig. 5c). Approximately 594 BPs, 55 CCs, and 24 MF categories were identified from these analyses. Of the BP identified through the Database for Annotation, Visualization and Integrated Discovery analyses, GO categories for activation of mitogen-activated protein kinase activity, positive regulation of gene expression, negative regulation of apoptotic process, G2/M transition of mitotic cell cycle, and positive regulation of cell proliferation were upregulated in the senescent hCPCs after prolonged rapamycin treatment (Fig. 5c, lower left panel). In contrast, GO categories for the apoptotic process, negative regulation of cell growth, the immune response, and positive transcription regulation were downregulated in the senescent hCPCs after prolonged rapamycin treatment (Fig. 5c, lower right panel). Considering the Kyoto Encyclopedia of Genes and Genomes (KEGG) pathway enrichment analysis, ten KEGG pathways were differentially expressed following prolonged rapamycin-treated hCPCs (Fig. 5d); herein, the cell cycle signaling pathway (35/124 transcripts overlapped *p*-value 2.91E − 14; Fig. 5d), PI3K-AKT signaling pathway (44/341 transcripts overlapped, adjusted *p*-value: 6.08E − 6; Supplementary Fig. S3), and JAK-STAT3 signaling pathway (19/158 transcripts overlapped, adjusted *p*-value: 0.005; Supplementary Fig. S3) were significantly associated with senescent hCPCs after prolonged rapamycin treatment. Interestingly, gene expression related to the JAK-STAT3 signaling pathways (*STAT3*, *PIM1*, and *BCL2*) was increased in senescent hCPCs after prolonged rapamycin treatment (Fig. 6a, b and Supplementary Fig. S3). Several studies have reported that *PIM1* is a cardioprotective gene^45,46^. Moreover, *PIM1* restores senescence in c-Kit^+^ hCPCs^47^. In a myocardial infarction swine model, the overexpression of *PIM1* had therapeutic engraftment effects in hCPCs^48^. Several studies have demonstrated that the JAK-STAT3 signaling axis is required for *PIM1* regulation in the heart. Specifically, p-STAT3 binds to the *PIM1* promoter and triggers its activation^49^. In addition, *STAT3* is a key regulator of cell-to-cell communication in the heart^50^ and the STAT3-PIM1 axis is essential for differentiating Sca1^+^ CPCs into endothelial cells^51^.

To determine whether the STAT3-PIM1 signaling axis affects senescent hCPCs after prolonged rapamycin treatment, we investigated the proliferation ability and age-related deterioration in senescent hCPCs by treating the cells with STAT3 and PIM1 inhibitors. The cell proliferation rates were decreased and the number of SA-β-gal^+^ cells were increased after treatment with STAT3 (Fig. 6c, d) and PIM1 (Fig. 6e, f) inhibitors in the senescent hCPCs after prolonged rapamycin treatment. In addition, the expression of both cyclin-dependent kinase inhibitors and senescence-associated markers, such as p21 and p16, were significantly increased after treatment with STAT3 and PIM1 inhibitors (Fig. 6g). Interestingly, the mRNA levels of the senescence-associated marker p53 were significantly decreased in the STAT3 inhibitor-treated group. The Pim1 inhibitor-treated group showed that p53 mRNA levels were significantly increased. These data demonstrate that the STAT3-PIM1 axis is involved in senescent hCPCs after prolonged rapamycin treatment.

### Effects of differentiation ability when a STAT3-Pim1 inhibitor is administered to rapamycin-treated hCPCs

**Fig. 7:**
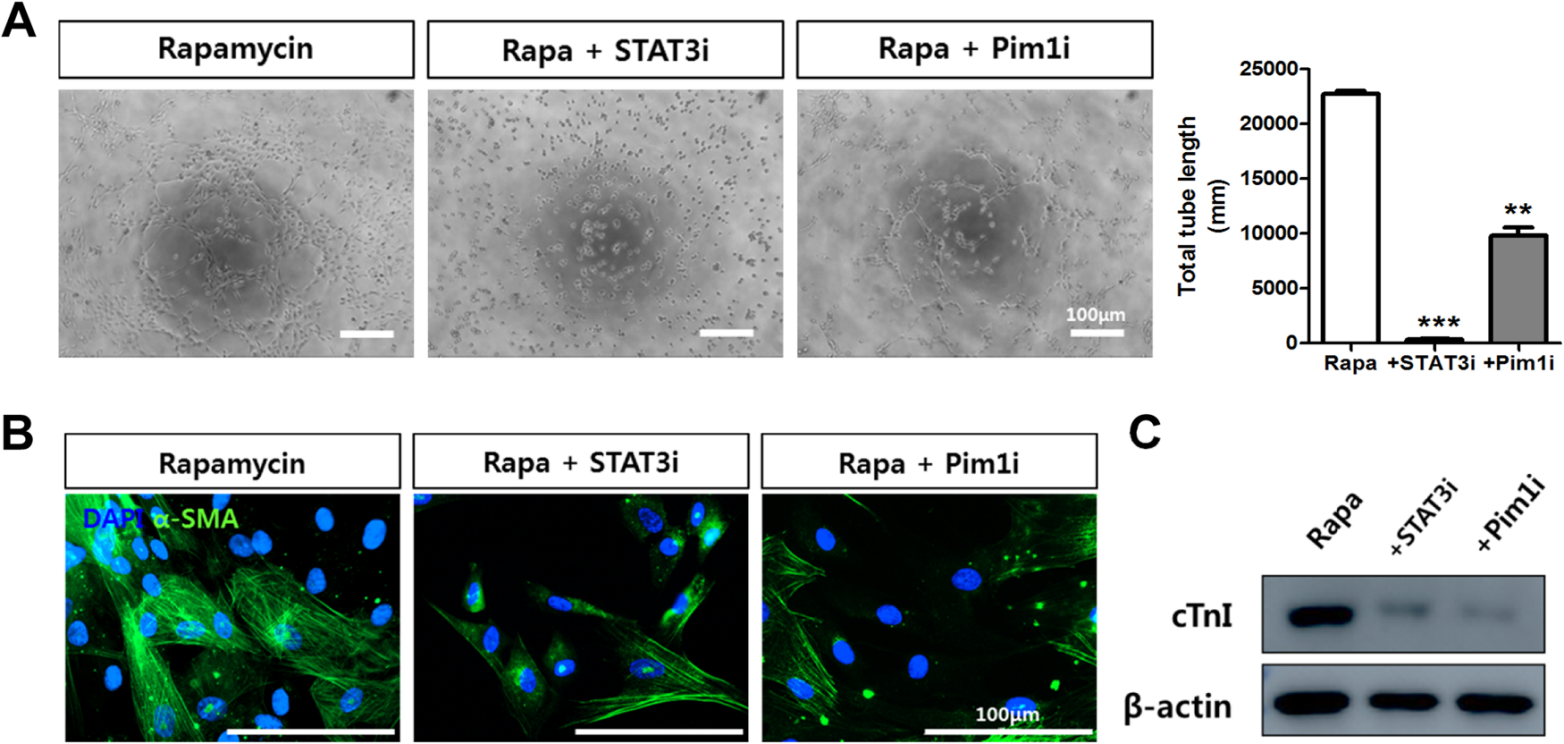
The STAT3-PIM1 axis affects the differentiation ability of rapamycin-treated hCPCs. **a** A STAT3 inhibitor (10 μM) and a Pim1 inhibitor (20 μM) were added to rapamycin-treated hCPCs, and Matrigel tube formation assays were examined. Data represent the mean ± SEM of three independent experiments (**p ≤ 0.01, *** *p* ≤ 0.001, vs. prolonged rapamycin-treated hCPCs). **b** Smooth muscle differentiation ability as analyzed by α-SMA staining after induced differentiation (scale bar = 100 μm). **c** Myogenic potential was analyzed by cTnI immunoblot assay after 28 days of induced differentiation. Abbreviation: STAT3i STAT3 inhibitor, Pim1i Pim1 inhibitor.

Finally, to determine whether the STAT3-Pim1 axis affects the differentiation ability of rapamycin-treated hCPCs, we conducted differentiation assays. A tube formation assay revealed that after treatment with a STAT3 inhibitor (10 μM), the total tube length significantly decreased (*p* < 0.001). The Pim1 inhibitor (20 μM)-treated group also revealed significantly decreased tube formation ability (Fig. 7a, *p* < 0.01). Next, we induced SMC differentiation using a STAT3 inhibitor of Pim1. SMC marker α-SMA-immunostained images showed that administering a STAT3 inhibitor or Pim1 inhibitor to rapamycin-treated hCPCs decreased differentiation ability (Fig. 7b). Myogenic differentiation potential also decreased in both the groups treated with STAT3 and Pim1 inhibition (Fig. 7c). Taken together, the STAT3-Pim1 axis affects not only senescence but also differentiation ability in rapamycin-treated hCPCs.

## Discussion

The predominant mechanisms of aging are related to the accumulation of senescent cells in tissues and cells^2^, including cardiomyocytes in the heart^5^. Moreover, hCPCs act as key regulators of cardiomyocyte homeostasis in the heart^14,15^ and contribute to the repair of damaged heart tissue during aging^16^. Senescent and dysfunctional hCPCs lead to premature cardiac aging and heart failure^13^.

**Fig. 8:**
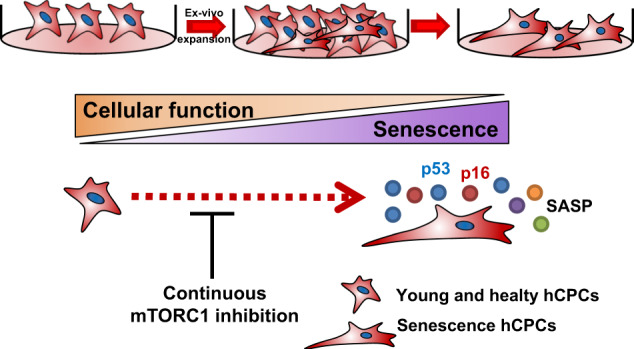
Prolonged rapamycin treatment attenuates replicative senescence and improves cellular function in hCPCs. We initially generated cellular replicative senescence in hCPCs and characterized the molecular and physiological phenotype of replicative senescence in hCPCs. Furthermore, pharmacological inhibition of mTOR by rapamycin can attenuate replicative cell senescence and improve cellular function in hCPCs.

In this study, we initially demonstrated that the effect of mTOR inhibition by rapamycin markedly improved cellular functions in hCPCs (Fig. 8). First, we found that rapamycin is a potential attenuating agent for replicative senescence in hCPCs. Second, we observed that rapamycin promotes the proliferation of senescent hCPCs. Third, we found that prolonged rapamycin treatment enhances the clonogenic potential and migration/differentiation ability in senescence hCPCs. Ultimately, we found that rapamycin not only inhibited mTOR signaling but also influenced the STAT3-PIM1 signaling axis in senescent hCPCs after prolonged rapamycin treatment.

hCPCs are essential for vascular repair in ischemia and are capable of differentiating into endothelial cells, SMCs, and cardiomyocytes^16^. On the basis of previously published data and our data, it has been shown that it is essential to obtain hCPCs with better quality and in higher quantity for the best clinical treatment of myocardial infarction^52,53^. In these studies, superior hCPCs were created during ex vivo expansion under hypoxic conditions to improve their quality and quantity. These cells reveal better qualities in terms of high proliferation ability, low chromosomal abnormality, resistance to oxidative stress, cell engraftment, and differentiation. In addition, hypoxic preconditioning during ex vivo expansion of hCPCs activates the ERK signaling pathway^53^, which is crucial for the regulation of various cellular processes, including proliferation, survival, differentiation, and development^54^. Under hypoxic conditions or hypoxic preconditioning, ERK activation promotes the bioactivity of hCPCs^53^ and promotes cardiac cell generation from the differentiation of human cord blood-derived mesenchymal stem cell^55^. In addition, recent studies reported that hypoxic preconditioning promotes cellular proliferation and osteogenic differentiation of human mesenchymal stem cells^56,57^, and increases endothelial cell generation from the differentiation of human bone marrow CD133+ cells^58^. Whether physiological changes such as hypoxic preconditioning affect cell differentiation and regulate specific lineage differentiation remains unclear. Nevertheless, this can be addressed in future studies, particularly with regard to molecular cascades and physiological conditions.

The mTOR signaling pathway efficiently regulates tissue homeostasis, including the heart. Rapamycin is an mTOR inhibitor and an autophagy inducer^33^. First, several studies reported that inhibition of mTORC1 by knockdown of RAPTOR promotes cardiac differentiation^59^; however, inhibition of mTORC2 via knockdown of its component RICTOR suppresses cardiac differentiation^60^. Prolonged rapamycin treatment inhibits the assembly of mTORC2^31^. Rapamycin can continuously promote cardiomyogenesis throughout the process of cardiac induction^33^. Second, the present literature on the autophagy signaling pathway indicates that it plays a crucial role in cell metabolism, proliferation, differentiation, and replicative senescence of stem cells^61–63^. A recent study suggests that doxorubicin (DOXO)-mediated hCPC toxicity is related to autophagy signaling and was attenuated by prolonged rapamycin treatment in hCPCs^14^. DOXO is widely used to treat solid tumors; however, its clinical use is limited by its side effects, such as cardiotoxicity and cardiomyocyte damage^64^. In addition, rapamycin recovers the autophagosome formation capacity of DOXO-treated hCPCs. These data suggest that the modulation of autophagy via inhibition of mTOR signaling by prolonged rapamycin treatment may hamper DOXO-mediated cardiotoxicity. Collectively, our data identify a crucial function for prolonged rapamycin treatment in hCPCs.

In summary, our data demonstrate the critical mechanism for prolonged rapamycin treatment in senescent hCPCs. We believe that our findings pave the way for further investigations of novel therapeutic interventions in senescent hCPCs, which will overcome the present obstacles in cardiac regeneration and repair by generating hCPCs of better quality.

### Supplementary information

supplemental materials
