# Author Correction: Pharmacological inhibition of mTOR attenuates replicative cell senescence and improves cellular function via regulating the STAT3-PIM1 axis in human cardiac progenitor cells

**DOI:** 10.1038/s12276-023-01127-5

**Published:** 2023-11-27

**Authors:** Ji Hye Park, Na Kyoung Lee, Hye Ji Lim, Seung taek Ji, Yeon-Ju Kim, Woong Bi Jang, Da Yeon Kim, Songhwa Kang, Jisoo Yun, Jong seong Ha, Hyungtae Kim, Dongjun Lee, Sang Hong Baek, Sang-Mo Kwon

**Affiliations:** 1 Laboratory of Regenerative Medicine and Stem Cell Biology, Department of Physiology, Medical Research Institute, School of Medicine, Pusan National University, Yangsan, 50612 Republic of Korea; 2 Research Institute of Convergence Biomedical Science and Technology, Pusan National University School of Medicine, Yangsan, 50612 Republic of Korea; 3 R&D Center for Advanced Pharmaceuticals & Evaluation, Korea Institute of Toxicology, Korea Research Institute of Chemical Technology, 141 Gajeong-ro, Yuseong-gu, Daejeon, 34114 South Korea; 4 Department of Thoracic and Cardiovascular Surgery, Pusan National University Yangsan Hospital, School of Medicine, Pusan National University, Yangsan, 50612 Republic of Korea; 5 Department of Convergence Medical Science, Pusan National University School of Medicine, Yangsan, 50612 Republic of Korea; 6 Laboratory of Cardiovascular Disease, Division of Cardiology, School of Medicine, The Catholic University of Korea, Seoul, 137-040 Republic of Korea

**Keywords:** Adult stem cells, Heart stem cells

Correction to: *Experimental & Molecular Medicine* 10.1038/s12276-020-0374-4, published online 09 April 2020

In this article the affiliation details for Hyungtae Kim were incorrectly given as ‘Department of Thoracic and Cardiovascular Surgery, Pusan National University Yangsan Hospital, Yangsan 50612, Republic of Korea’ but should have been ‘Department of Thoracic and Cardiovascular Surgery, Pusan National University Yangsan Hospital, School of Medicine, Pusan National University, Yangsan 50612, Republic of Korea’.

The authors apologize for any inconvenience caused.

The original article has been corrected.

