# Supplementary material for: Pharmacological inhibition of mTOR attenuates replicative cell senescence and improves cellular function via regulating the STAT3-PIM1 axis in human cardiac progenitor cells
Source: Exp Mol Med. 2020 Apr 9;52(4):615–28. doi: 10.1038/s12276-020-0374-4 (PMC7210934; doi:10.1038/s12276-020-0374-4)
Supplement: Supplementary file 1 — supplemental materials [file 12276_2020_374_MOESM1_ESM.docx]

**Supplementary Materials.**

**Supplymentary Table1. Primers for qRT-PCR.**

| Gene | Sense | Anti-sense | Reference |
| --- | --- | --- | --- |
| P16 | CCCCGATTGAAAGAACCAGAGA | ACGGTAGTGGGGGAAGGCATAT | ^1^ |
| P21 | CCGCCCCCTCCTCTAGCTGT | CCCCCATCATATACCCCTAACACA | ^1^ |
| P53 | CCGGCGCACAGAGGAAGAGA | TGGGGAGAGGAGCTGGTGTTGT | ^1^ |
| CENP-A | ACGCCTATCTCCTCACCTT | TGGCTGAGCAGGAAAGAC | ^2^ |
| Pim1 | TGCCATTAGGCAGCTCTCCCCA | GCGGCTTCGGCTCGGTCTACT | ^3^ |
| STAT3 | GCTTCTCCTTCTGGGTCTGGC | CCTCCTTCTTTGCTGCTTTCACT | ^4^ |
| Bcl2 | TTTTTCTCCTTCGGCGGG | GGTGGTCATTCAGGTAAGTGGC | ^5^ |
| IL-1A | AATGACGCCCTCAATCAAAG | TGGGTATCTCAGGCATCTCC | ^6^ |
| IL-6 | TACCCCCAGGAGAAGATTCC | TTTTCTGCCAGTGCCTCTTT | ^6^ |
| OCT4 | CAGTGCCCGAAACCCACAC | GGAGACCCAGCAGCCTCAAA |  |
| NANOG | CAGAAGGCCTCAGCACCTAC | ATTGTTCCAGGTCTGGTTGC |  |
| KLF4 | CAAAGAGTTCCCATCTCAAG | AAAAATGCCTCTTCATGTGT |  |
| FN1 | AGGAAGCCGAGGTTTTAACT | AGGACGCTCATAAGTGTCAC |  |
| HGF | GCAATTTTTGGTTTGGCTGT | CCTGCCCACAGCATATAGGT |  |
| IGF1 | TGGATGCTCTTCAGTTCGTG | TGGTAGATGGGGGCTGATAC |  |
| CNN1 | AGGCTCCGTGAAGAAGATCA | CCACGTTCACCTTGTTTCCT |  |

Reference

1 Zhou, N. *et al.* SIRT1 alleviates senescence of degenerative human intervertebral disc cartilage endo-plate cells via the p53/p21 pathway. *Sci Rep* **6**, 22628, doi:10.1038/srep22628 (2016).

2 Tomonaga, T. *et al.* Overexpression and mistargeting of centromere protein-A in human primary colorectal cancer. *Cancer Res* **63**, 3511-3516 (2003).

3 Mohsin, S. *et al.* Rejuvenation of human cardiac progenitor cells with Pim-1 kinase. *Circ Res* **113**, 1169-1179, doi:10.1161/CIRCRESAHA.113.302302 (2013).

4 Yang, M. *et al.* Role of the JAK2/STAT3 signaling pathway in the pathogenesis of type 2 diabetes mellitus with macrovascular complications. *Oncotarget* **8**, 96958-96969, doi:10.18632/oncotarget.18555 (2017).

5 Bussolati, B. *et al.* Isolation of renal progenitor cells from adult human kidney. *Am J Pathol* **166**, 545-555, doi:10.1016/S0002-9440(10)62276-6 (2005).

6 Laberge, R. M. *et al.* MTOR regulates the pro-tumorigenic senescence-associated secretory phenotype by promoting IL1A translation. *Nat Cell Biol* **17**, 1049-1061, doi:10.1038/ncb3195 (2015).

**
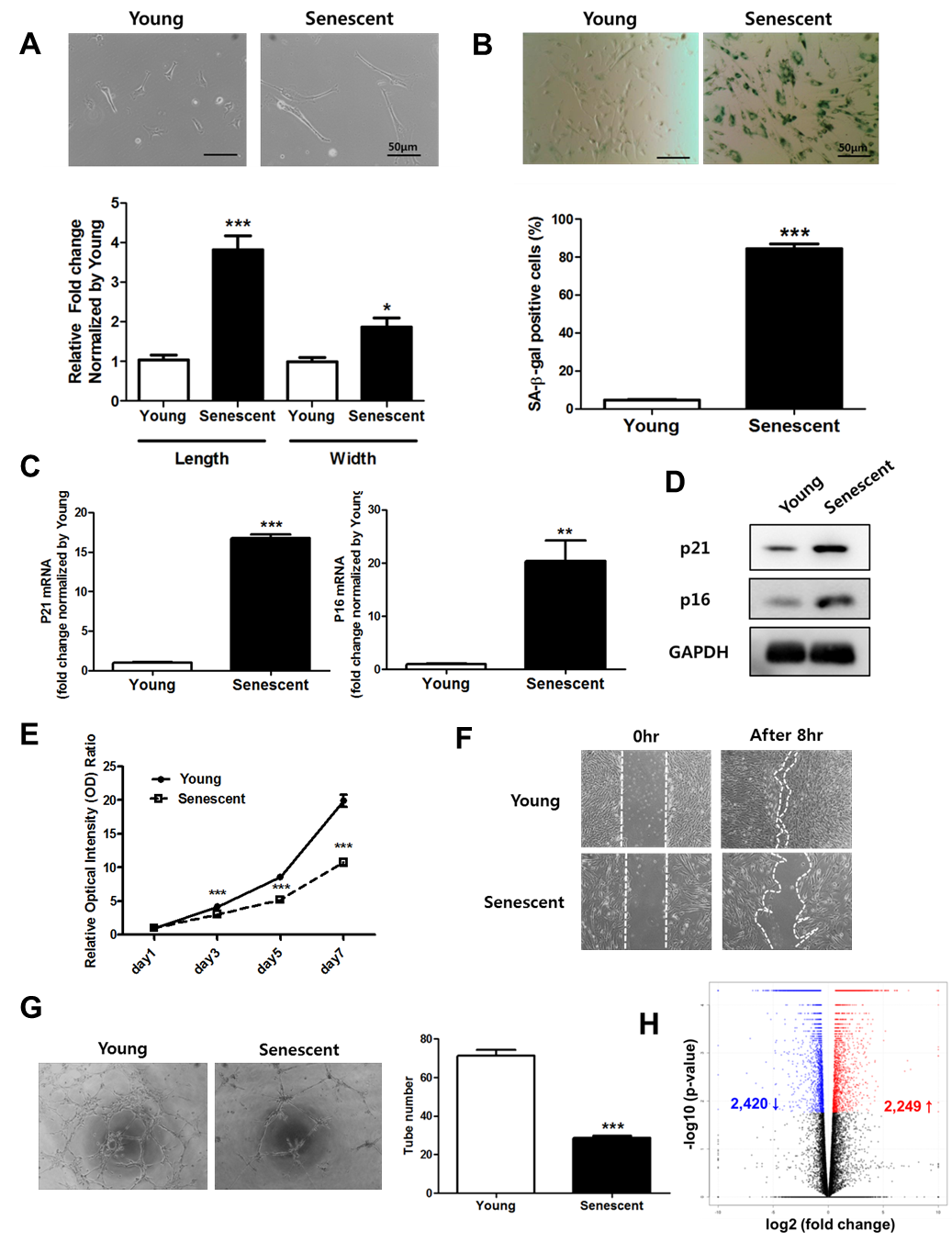
**

**Figure S1. Replicative senescence hCPCs characterization.** (A) The Morphological analysis between young and senescent hCPCs (B) SA-b-gal positive assay between young and senescent hCPCs. (C and D) Expression of Senescence marker p21 and p16 in the senescent hCPCs. (E) Cell proliferation ability was determined from the senescent hCPCs. (F) Cell migration assay was examined in the senescent hCPCs by the wound scratch method. (G) Tube forming ability of the senescent hCPCs. (H) Gene expression of the senescent hCPCs. (2249 genes were upregulated and 2420 genes were downregulated in replicative senescence hCPCs). cut off: q-value < 0.05.

**
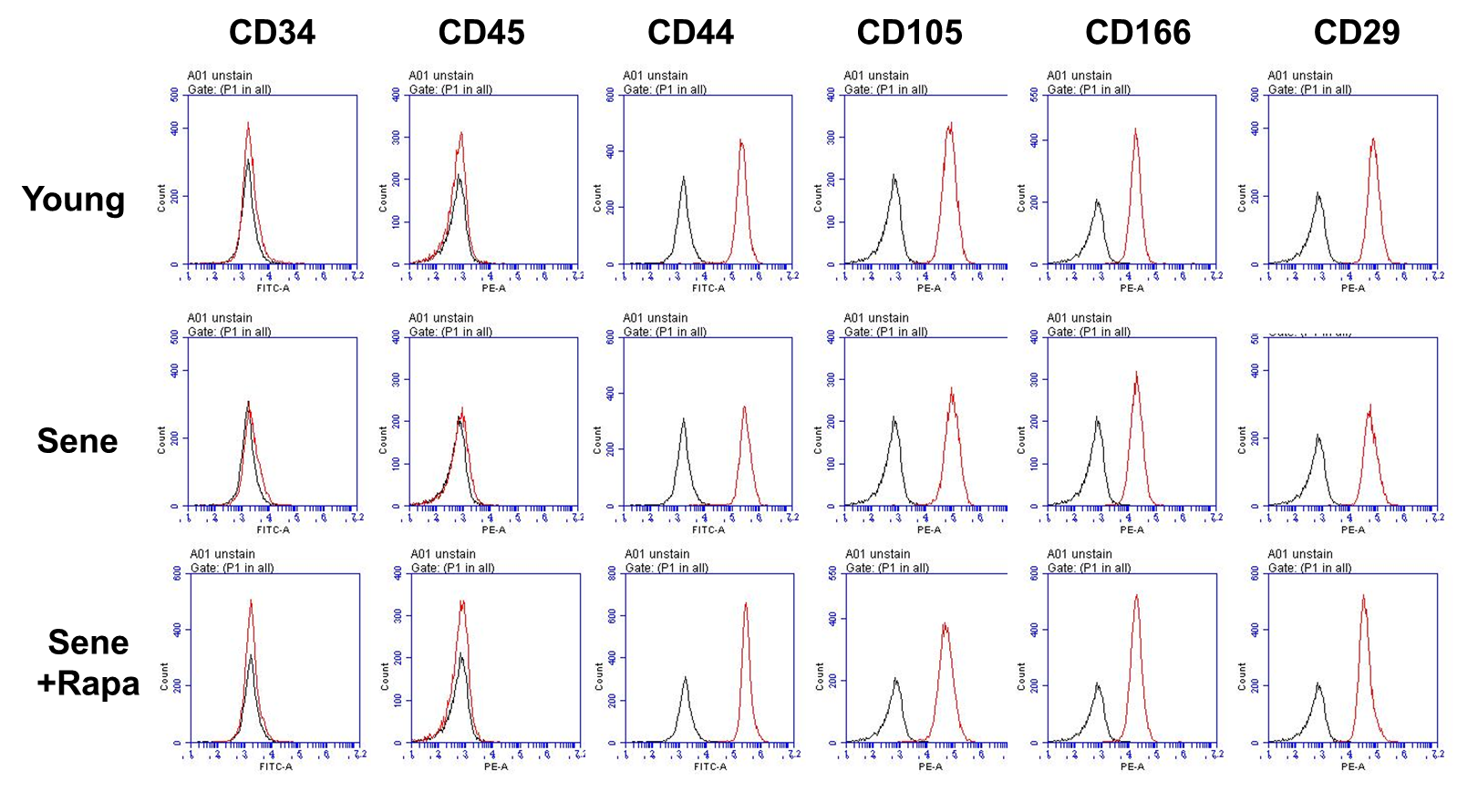
**

**Figure S2. FACS analysis on hCPCs.** Hematopoietic lineage markers CD34 and CD45 were negatively expressed in three groups of hCPCs. The expression of CD44, CD105, CD166, and CD29 was differentially expressed in young, senescence, and chronically treated rapamycin hCPCs.

**
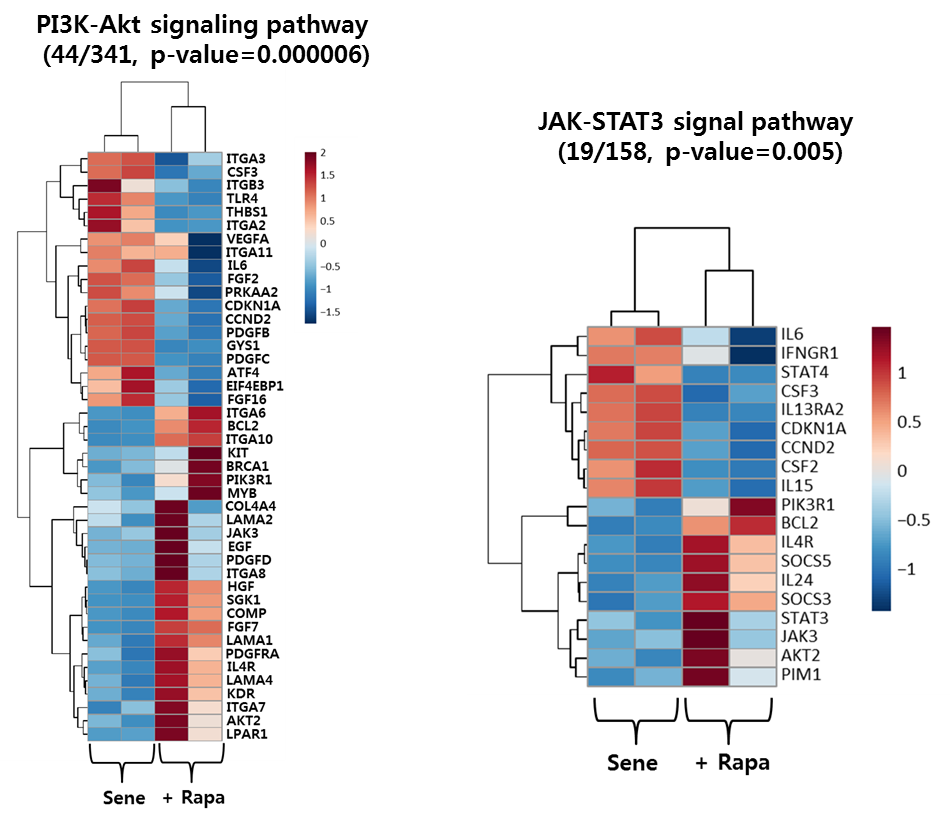
**

**Figure S3. Heat map of PI3K-AKT and JAK-STAT signaling pathways.** Differentially expressed genes of the PI3K-Akt signaling (left panel) and JAK-STAT signaling (right panel) pathway in senescence hCPCs before and after chronic rapamycin treatment were presented. Normalized log10 FPKM, *p*-value: 0.005.
